# Forecasting COVID19 parameters using time-series: KSA, USA, Spain, and Brazil comparative case study

**DOI:** 10.1016/j.heliyon.2022.e09578

**Published:** 2022-06-02

**Authors:** Souad Larabi-Marie-Sainte, Sawsan Alhalawani, Sara Shaheen, Khaled Mohamad Almustafa, Tanzila Saba, Fatima Nayer Khan, Amjad Rehman

**Affiliations:** a Department of Computer Science, College of Computer and Information Sciences, Prince Sultan University, Riyadh 11586, Saudi Arabia; b Artificial Intelligence Data Analytics (AIDA) Lab, College of Computer and Information Sciences, Prince Sultan University, Riyadh 12435, Saudi Arabia; c Department of Information Sciences, College of Computer and Information Sciences, Prince Sultan University, Riyadh 11586, Saudi Arabia

**Keywords:** COVID-19, Forecasting, Drift, Exponential smoothing, Holt, Linear regression, Time-series

## Abstract

Many countries are suffering from the COVID19 pandemic. The number of confirmed cases, recovered, and deaths are of concern to the countries having a high number of infected patients. Forecasting these parameters is a crucial way to control the spread of the disease and struggle with the pandemic. This study aimed at forecasting the number of cases and deaths in KSA using time-series and well-known statistical forecasting techniques including Exponential Smoothing and Linear Regression. The study is extended to forecast the number of cases in the main countries such that the US, Spain, and Brazil (having a large number of contamination) to validate the proposed models (Drift, SES, Holt, and ETS). The forecast results were validated using four evaluation measures. The results showed that the proposed ETS (resp. Drift) model is efficient to forecast the number of cases (resp. deaths). The comparison study, using the number of cases in KSA, showed that ETS (with RMSE reaching 18.44) outperforms the state-of-the art studies (with RMSE equal to 107.54). The proposed forecasting model can be used as a benchmark to tackle this pandemic in any country.

## 1 Introduction

The current pandemic, COVID-19, has its first detection in December 2019 in Wuhan, China. It was declared as Pandemic by the World Health Organization on 04 May 2020 and is still affecting people globally.

To fight with the pandemic, it is crucial to forecast the spread of the disease by considering not only the number of cases but also the number of death and recoveries. The accurate and reliable forecasting results for a given period can support the health and government entities to design their health strategies to address the expected consequences of the pandemic.

This real-world problem can be seen as demand forecasting which is a predictive analysis that estimates the customer demand to enhance supply decisions and business management. In this study, the customer demand represents the COVID19 parameters (the number of cases, deaths, and/or recovered), whereas the supply stands for the health sector and government entities. Demand forecasting plays a crucial role in decision making. The efficiency of a decision depends on the forecasted results.

Machine Learning (ML) models and statistical analysis are more powerful tools to predict the severity of the outbreak and identify at-risk populations across the countries and regions. Recently, different mathematical and Machine Learning-based forecasting models were proposed to forecast the number of cases and determine its impact, globally and for specific countries such as the USA, Brazil, China, Italy, Spain, India, and Malaysia. However, to the best of our knowledge, two studies have been carried out to forecast the number of COVID-19 cases in KSA [1, 2].

In this study, the statistical time-series techniques are applied to provide the accurate and reliable forecasting results for the number of confirmed cases and the number of deaths in KSA, USA, Spain, and Brazil. The use of time-series in forecasting infectious diseases has been early studied and recommended by different researchers (e.g. [3]).

A time-series is a sequence of values ordered by time. It is assumed to be stationary. In other words, it must not rely on the time at which the time-series is perceived.

To fulfill this study, four forecasting techniques (Drift, Simple-Exponential-Smoothing (SES), one variant of Exponential-Smoothing (ETS), and Holt) were used. These techniques have proved their success in forecasting different diseases [4, 5], including COVID-19 parameters [6].

Drift is one of the simplest methods. It is usually considered a benchmark [7]. Drift is one variant of the Naive forecasting method. It permits the forecasting results to raise or diminish across time. This is performed by considering the average change in the whole data instead of the total change over time. ETS belongs to the Exponential Smoothing family algorithms. It incorporates three terms including Error, Trend, and Season (why its name ETS). Each term can be combined using the addition or the multiplication or dropped from the model [7]. SES is one of the Exponential Smoothing family methods [7]. SES is dedicated to forecasting data with no distinct seasonal or trend. Therefore, it was employed in this study. Holt technique [7] was derived from the Simple Exponential Smoothing (SES) to promote the forecasting of data possessing trends.

Unlike the existing studies [1, 2] that used the Auto-Regressive Integrated Moving average (ARIMA), the Auto-Regressive Moving average (ARMA), and the Logistic Growth to forecast the number of confirmed cases in KSA, this study aims at providing an efficient forecast model of the confirmed cases for different countries using the aforementioned methods. Moreover, it also provides the forecast of the number of deaths for KSA. Note that, these methods were not involved in recently published studies to forecast the COVID19 parameters.

The contribution of the present work is as follows.

• Show the effectiveness of the Exponential Smoothing techniques in forecasting the spread of COVID19 disease.
• Forecast the COVID19 parameters using only the past confirmed cases/deaths numbers without requiring additional factors.
• Develop an effective model, that outperforms the existing models, to forecast the COVID-19 parameters
• Use the developed model to forecast the confirmed cases and deaths in any country and at any time.
• Suggest the developed models to forecast the spread of any disease.

The experiments passed through five main phases. Firstly, the time-series stationary was validated using well-known techniques and tests. Then, the residuals of each model were investigated to ensure that the models can be applied to forecast new values. After that, the best forecasting model was selected based on the Root Mean Square Error (RMSE). Later, the best model was validated using four evaluation measures (RMSE, Mean Absolute Error (MAE), Mean Percentage Error (MPE), and Autocorrelation of errors at lag 1 (ACF1)). Finally, the numbers of cases (respectively the number of deaths) are successfully forecasted using the prediction intervals (85% and 90%) for each country (respectively for KSA) for June 2020. The research findings prove that the number of cases/deaths was successfully forecasted. The comparison study showed that the proposed models outperformed the model proposed in the related works.

This article is organized as follows. Section 2 discusses the recent existing studies. Section 3 describes the methodology. Section 4 encompasses the experimentation. Section 5 concludes this study.

## 2 Related work

Recently, numerous research studies have been done to model COVID-19 with the goal of better understanding of the pandemic. Most of these studies are focused on predicting the disease based on a patient's medical diagnosis. For example, in [8], the authors developed a new system called the “Gui Covid-19 prediction desktop tool”. The proposed system automatically detects the infection through the Chest X-Ray images using the Convolutional Neural Network (CNN). The authors in [9] also used X-Ray images to detect whether the patient is infected or not. They applied the Scatter Wavelet Transform for image segmentation and preprocessing and then the Dense Deep Neural Network for the prediction. Moreover, in [10] the authors focused on the same data type (X-Ray) and applied a modified version of CNN called a Siamese CNN model to automatically detect the COVID-19 infected patients.

The prediction of infected patients based on the medical diagnosis is mainly performed using Machine and Deep Learning. This methodology is generally based on X-Ray images, which is very different from forecasting the number of future infected patients using numerical data. The researchers are still using the time-series to handle this matter. In [4], the authors forecasted the numbers of COVID-19 confirmed and recovered cases worldwide using Autoregressive time-series models based on two-piece scale mixture normal distributions. The proposed technique performed well and outperformed the existing models. The authors in [5] studied the performance of different time-series methods to predict the number of COVID-19 active cases. The statistical methods outperformed the Deep Learning (DL) methods. The authors in [11] used the Genetic based Programming model to forecast the behavior of COVID19 spreading in India. The obtained results were highly reliable. The complex network methods have also been used to forecast the spreading of the outbreak [12].

The authors in [13] used Linear Regression (LR), Multilayer perceptron, and Vector autoregression to predict the pace of the spreading of COVID19 in India. Forecasting the confirmed and death cases using the exponential smoothing family was presented [6]. The results indicated a significant increase in the spread of the disease. The mortality rate caused by COVID-19 [14] was also investigated using the Patient Information Based Algorithm (PIBA) to estimate the death rate among COVID-19 infected patients in China. The death rate ranges from 0.75% to 3% and may decrease in the future which is consistent with the real records of death cases. The authors in [15] suggested three DL models based on Recurrent Neural Network (RNN) named Stacked Long-Term Memory (LSTM), Convolution LSTM, and Bi-directional LSTM to forecast the COVID19 number of cases and deaths in India and the US. The results showed that the Convolution LSTM is the best model in forecasting both parameters for US and India.

In KSA, the authors in [1] used different models (Autoregressive Model, Moving Average, a combination of ARMA and ARIMA). They predicted that the new cases reach up to 7668 per day and over 127,129 cumulative cases by June 2020. Also, the authors in [2] used the Logistic Growth and the Susceptible-Infected-Recovered. They estimated the total number of confirmed cases to be around 69,000 in June2020. In [16], the authors applied SutteARIMA method to forecast the number of cases in the US. The estimated result was around 3 million cases from 26 June to 6 July 2020. The Mean Absolute Percentage Error (MAPE) achieved 0.539.

The time-series along with the basic statistical methods have also been used in forecasting other diseases. The authors in [17] compared the use of Neural Networks (NN) with the traditional seasonal ARIMA model for human brucellosis in mainland China. The study showed that the use of recurrent NN achieved much higher forecast accuracy, especially for non-linear time-series data. The authors in [3] used the Seasonal ARIMA model which succeeded to predict an annual periodicity/seasonal variation of hand-foot-mouth disease in China. The author in [18] applied time-series regression models to show the dependence between infectious diseases and weather conditions.

In the following, some studies related to disease prediction-based Machines and Deep Learning were presented. The authors in [19] presented a review of using ML for the prediction of Chronic Diseases. They reviewed 453 papers published between 2015 and 2019. They concluded that the most applicable and used ML models for such diagnosis were SVM and Logistic Regression (LR). The authors in [20] addressed the issue of missing data by proposing a regression-correlation combination (RCC) data imputation technique. They also studied the efficiency of the proposed labeling for the prediction of schistosomiasis disease density using Naive Bayes (NB), Support Vector Machine (SVM), J48 decision tree and Multi-Layer Perceptron (MLP) methods. The authors in [21] compared different supervised learning algorithms to perform prediction on a single disease. They found that SVM and the Naive Bayes algorithm were the most used algorithms. They also found that the algorithm that yielded the best accuracy in this context is the Random Forest (RF).

Moreover, in [22], the authors found that the CNN had better accuracy and less time and memory requirements than K-Nearest Neighbor (KNN) for disease prediction. Big data analysis has been also considered in [23] where three methods for the prediction of infectious disease spreading were evaluated. The authors concluded that DL techniques were the most stable. The LSTM models were more accurate than ARIMA. The authors in [24] presented a decision support system to classify and predict multiple diseases from medical data. They used Naive Bayes and J48 algorithms to analyze unseen patterns and relations in patients’ records. In [25], a model of big data for disease prediction was presented. The authors proposed a new CNN based on a multimodal prediction algorithm for regional disease risk prediction. They worked on structured and unstructured real-life hospital data and achieved 94.8% prediction accuracy.

Also, in [26], the authors presented a modified Bayesian Networks modeling and assessment methods for censored observations that have time-to-event relationship to predict cardiovascular risk from health data. Their proposed model outperformed the commonly used regression-based approach for time-to-event health data. The authors in [27] compared different ML algorithms such as Naive Bayes, Decision trees, K-Means, KNN and SVM for early diagnosis of diabetes mellitus. They tested the methods on PIMA Indian diabetes dataset. They suggested the most used algorithms and proposed recommendations for the least used algorithms. The authors in [28] proposed Active patient Risk Prediction (ARP) using active learning on medical data. The aim was to answer queries related to the similarities between patients that are difficult to answer by medical doctors.

## 3 Proposed methodology

**Figure 1:**
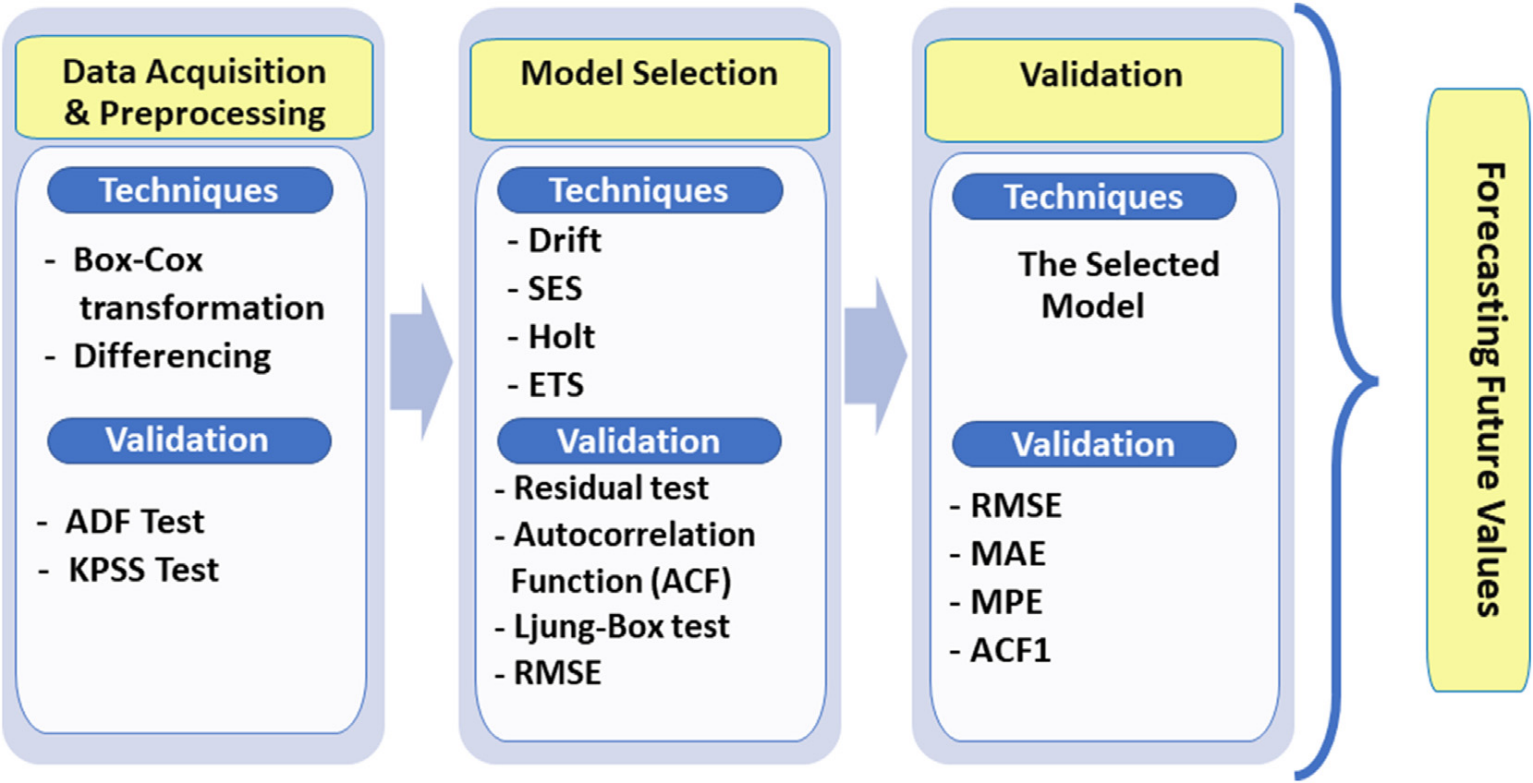
The overall methodology pipeline.

The proposed strategy is displayed in Figure 1. The first stage is a data acquisition and preprocessing. Starting with collecting and combining the concerned COVID-19 data (the numbers of cases, deaths, recovered) for KSA and the aforementioned countries and ending with applying several preprocessing techniques. The dataset was represented as a time-series and is further processed to make it stationary. Box-Cox Transformation and the differencing technique are used. The Augmented Dickey-Fuller (ADF) test and Kwiatkowski–Phillips–Schmidt –Shin (KPSS) test [7] are applied to check for data time-series stationary.

In the second stage, the forecasting techniques (Drift, SES, Holt, ETS) were used to find the best model for predicting future values. The models are validated using Residual's test, Auto correlation Function (ACF), and Ljung-Box test.

In fact, each dataset was divided into two sets, training, and testing. The training set was used for parameter setting and the selection of the best statistical model. In this case, two models, Holt and SES, require parameter setting. The 10-CV was applied to the training set to select the best parameters’ values for these models. After finding the best values, they were used (in the training model) to select the best statistical model.

The best model is selected based on:

1) The result of the ACF indicating that the residuals of the model are not correlated.
2) The Residual test indicating that the residuals are following a Normal distribution with constant variance and null means.
3) The highest P-Value calculated from the Ljung-Box test indicating that the residuals don't possess useful information required when forecasting future values.
4) The lowest Root Mean Square Error (RMSE).

The third stage involves the validation of the selected model using the testing set. Four evaluation measures were used to confirm the effectiveness of the selected model as described in Figure 1.

• The Root Mean Square Error (RMSE): measures the error resulting from the forecast points using the actual data points.
• The Mean Absolute Error (MAE): evaluates the accuracy of the model (when data is continues). The difference between the RMSE and the MAE indicates the variation in the individual errors.
• The Mean Percentage Error (MPE): indicates the difference between the forecasted and actual values. If the MPE is positive (resp. negative), then the forecast points are greater (resp. lesser) than the actual points.
• The Autocorrelation of errors at lag 1 (ACF1): indicates the correlation between the future points and the data points in the time-series.

The smaller the RMSE, MAE, and MPE values, the closer forecasted and actual points are.

Finally, inducing facts and results is the last stage where insights and accurate conclusions were gathered from the forecasting results.

## 4 Experimentation

The dataset was collected from (https://www.ecdc.europa.eu/en/publications-data) for KSA, Spain, US, and Brazil. In the following, two main experiments were conducted. The first experiment was to predict the number of cases for the four aforementioned countries. While the second experiment is to predict the number of deaths for KSA. Both experiments aim to show the efficiency of the proposed forecasting models. For the first experiment, the choice was based on countries with a high number of infected patients. The number of COVID 19 cases (resp. deaths) for each country (resp. for KSA) is represented using a time-series. As discussed in the methodology section, four forecasting methods (Drift, SES, Holt, and ETS) were tested to determine the best forecasting model. The experiments were performed using the R programming language (version 3.6.1).

### 4.1 Forecasting the number of cases in KSA

Table 1 shows the details of the KSA dataset, the minimum and maximum values, the total value, the mean, and the standard deviation. It also includes the size of the training and testing sets.

**Table 1:** KSA – dataset details.

Total	Min	Max	Mean	Standard Deviation	Training set size	Testing set size
108788	1	3369	1087.88	997.715	90	10

**Figure 2:**
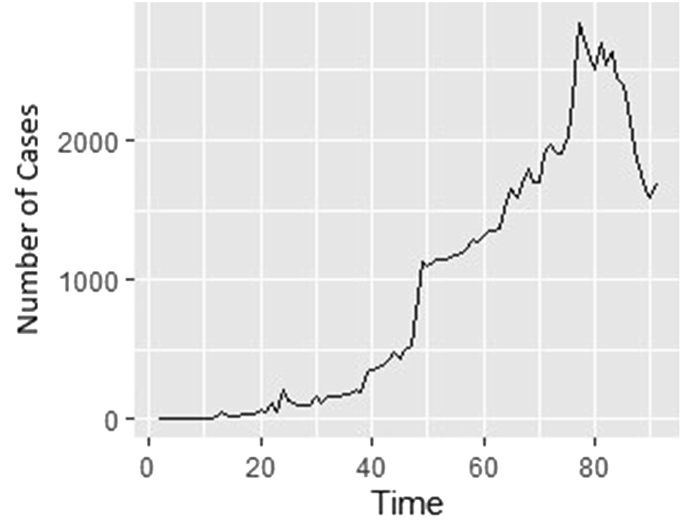
KSA: the time-series representing the number of cases (from March 2 to May 30). The Y-axis represents the number of cases and the X-axis stands for the time (the number of days).

The training set contains the number of cases starting from March 02to May 30. Figure 2 displays the time-series representing the training set. As seen, it has a trend and it is not stationary, the mean isn't null (= 928.9). The P-Value of ADF and KPSS are equal to 0.99 (>0.05) and 0.01 (<0.05) respectively which confirms the non-stationary of the time-series.

**Figure 3:**
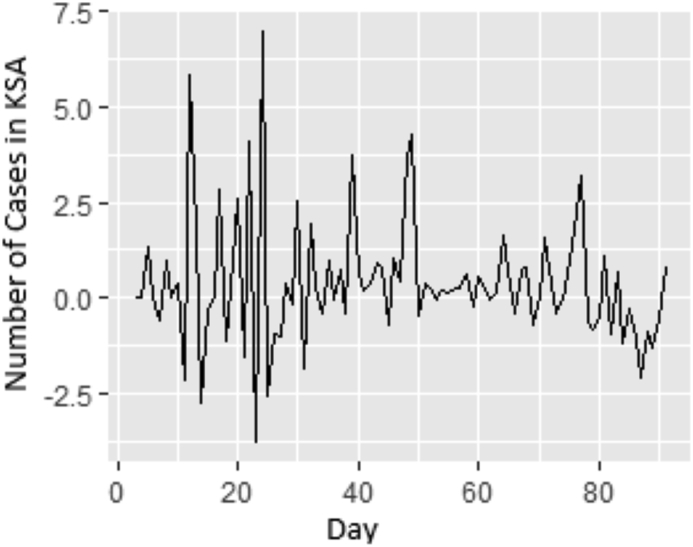
KSA: the differenced time-series for the number of cases (from March 2 to May 30). The Y-axis represents the number of cases and the X-axis stands for the time (the number of days).

After applying the Box-Cox transformation (λ = 0.3484489) and differencing the time-series, the new transformed time-series is stationary (see Figure 3) based on the results yielded by ADF and KPSS tests (P-Value of both tests achieved 0.02321 (<0.05) and 0.1 (>0.05) respectively).

**Figure 4:**
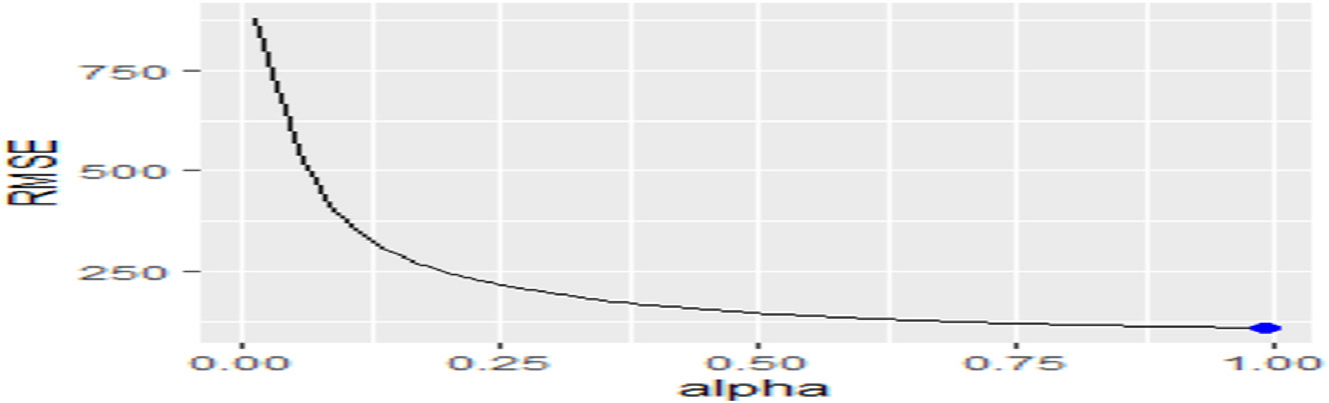
KSA – Parameter setting for SES using the number of cases. The Y-axis represents the RMSE values and the X-axis stands for the different values of alpha.

**Figure 5:**
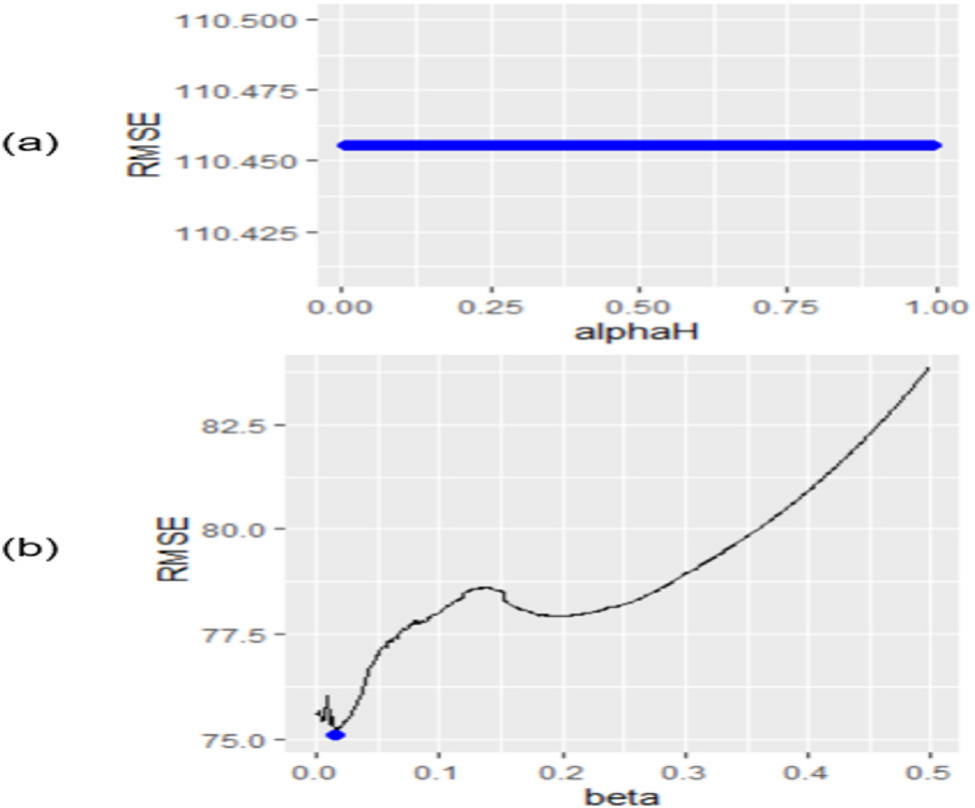
KSA – Parameter setting for Holt using the number of cases. The Y-axis represents the RMSE values (in both panels (a) and (b)) and the X-axis stands for the values of alphaH (in panel (a)) and beta (in panel (b)).

Note that SES and Holt require parameter settings. For that, 10-CV was applied in the training set. For SES (resp. Holt), the parameter α (resp. αH and β) was investigated to find the best value ranging between [0.01, 0.99] (resp. [0.01, 0.99] and [.0001, .5]) that minimizes the RMSE. Figures 4 and 5 display the minimum value of α, αH and β respectively. The best value of each parameter is α = 0.99, αH = [0.01,0.1] (the best values yielding the same RMSE value) and β = 0.0141.

**Figure 6:**
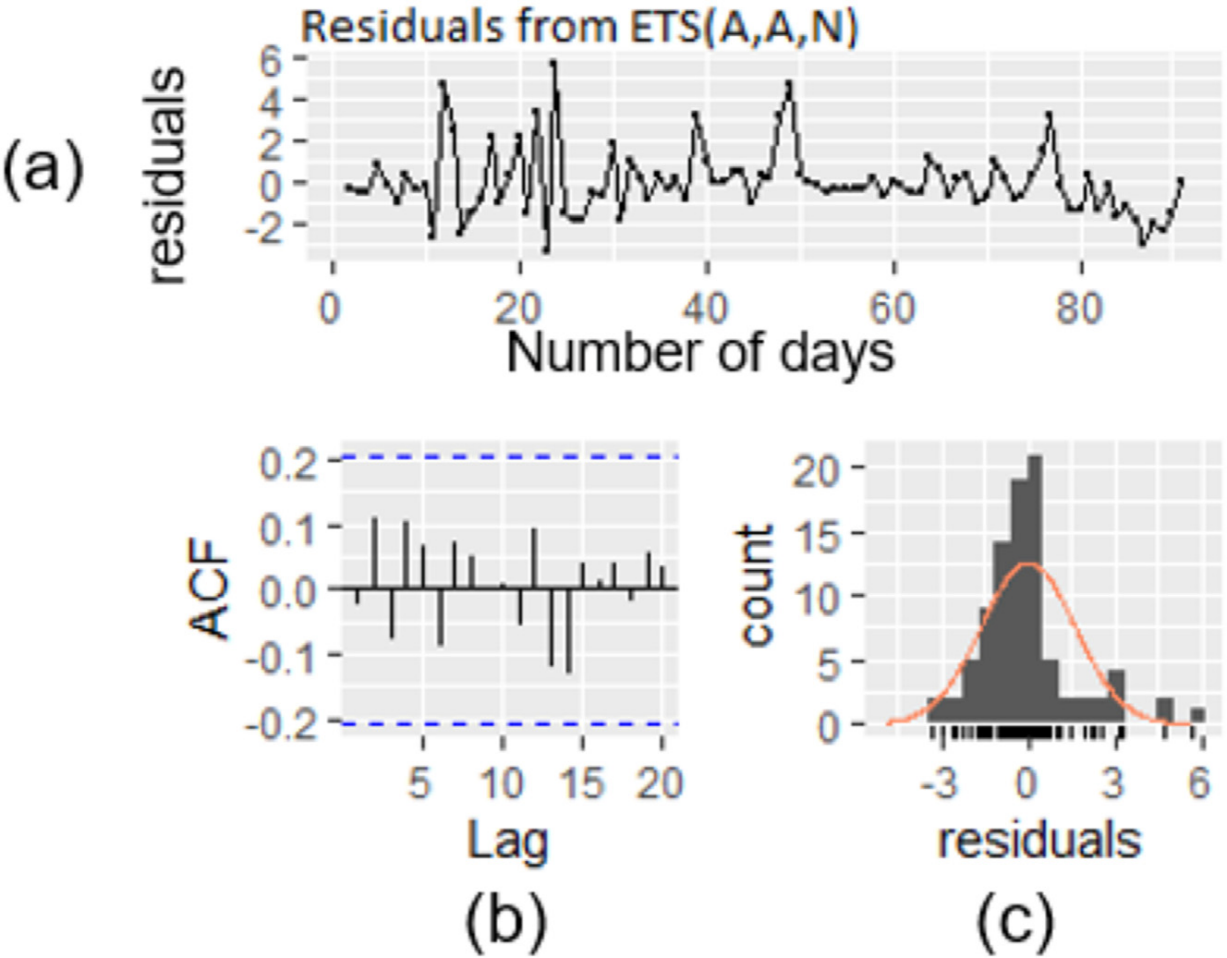
KSA Dataset – Residuals test for ETS forecasting model (from March 2 to May 30). The Y-axis represents the residuals values (in panel (a)), the ACF values (in panel (b)), and number count (in panel (c)). While the X-axis indicates the number of days (in panel (a)), the lags (in panel (b)), and the residuals (in panel (c)).

In order to validate each forecasting model, the residuals were investigated using ACF. The residuals of the four models are uncorrelated and follow the Normal distribution. Figure 6 displays the residuals (up), the ACF (bottom left), and the normal distribution of the residuals (bottom right) of the ETS model. To save space, only the ETS model was presented. To select the best model, Table 2 points out the P-Value of each model (calculated from the Ljung-Box test), and the RMSE. The P-Values of the four models are high. The SES, holt and ETS models successfully fit the data with a percentage between 58% and 79%. ETS and Holt yielded the lowest RMSE rate for both the training and the residuals. This is because the corresponding forecasts are based on the model that fitted to the entire data set. Consequently, the ETS model is selected.

**Table 2:** KSA - RMSE values from the training set and the residuals using the four forecasting methods.

RMSE	Drift	SES	Holt	ETS
P-Value	0.1115	0.7906	0.6521	0.5812
Training	430.1558	339.5058	20.8855	18.4361
Residuals	1.6716	1.6898	1.6085	1.6015

Table 3 displays the number of cases forecasted using ETS. It encompasses the date, the current number of cases, the forecasted values from May 31to June 09, 2020, and the lower and higher limits of 80% and 95% prediction intervals respectively. The forecasted values should always be accompanied by the prediction intervals because they cannot precise the uncertainty in the forecasts. These intervals express how accurate the forecasts are.

**Table 3:** KSA - The number of cases forecasted using ETS (May 31 to June 09).

Date	Real	Forecast	Lo 80	Hi 80	Lo 95	Hi 95
31/5/20	1877	1738	1473	2014	1348	2176
01/6/20	1881	1794	1459	2147	1305	2359
02/6/20	1869	1851	1454	2271	1276	2529
03/6/20	2171	1909	1456	2392	1257	2692
04/6/20	1975	1969	1462	2511	1244	2852
05/6/20	2591	2029	1472	2628	1235	3010
06/6/20	3121	2090	1484	2746	1230	3168
07/6/20	3045	2152	1498	2863	1227	3325
08/6/20	3369	2216	1514	2981	1228	3482
09/6/20	3288	2281	1532	3099	1230	3640

**Figure 7:**
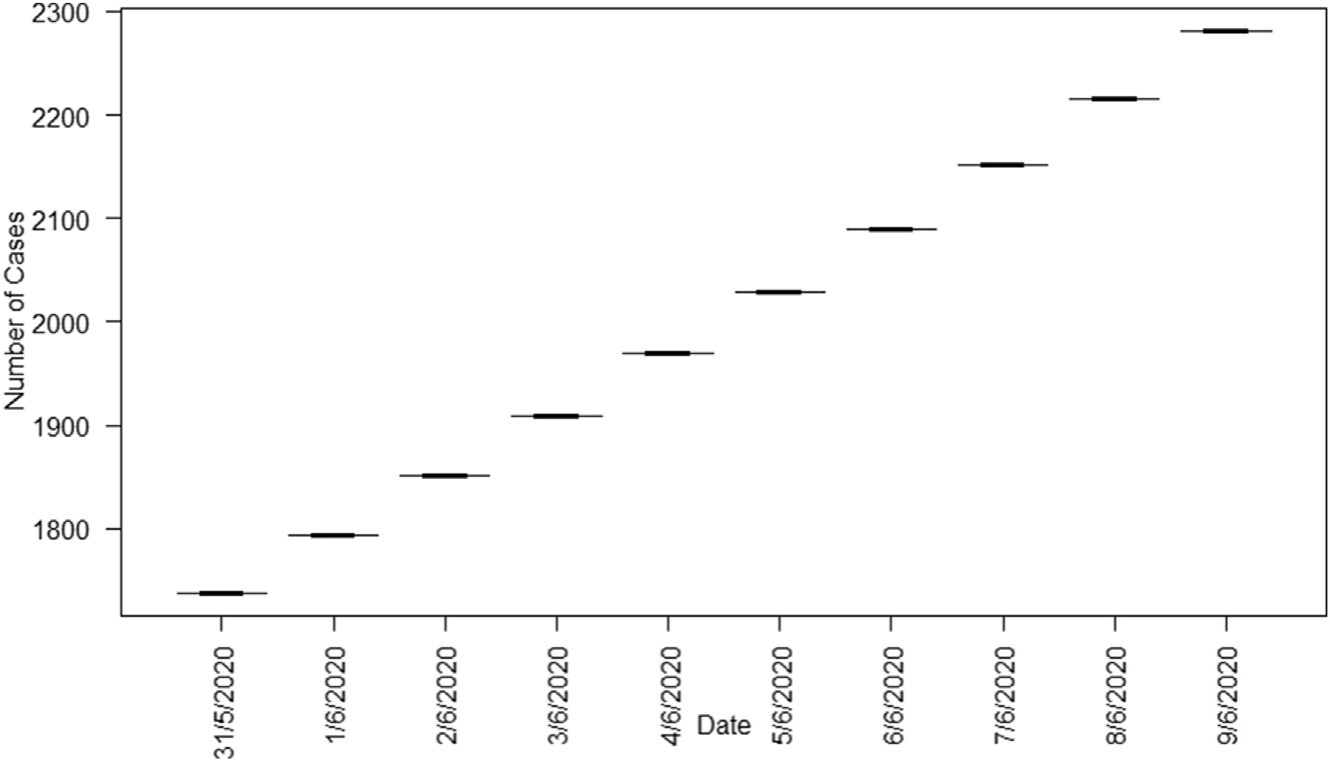
KSA – Boxplot of the Number of COVID19 cases forecasted using the ETS method between May 30 – June 9. The Y-axis represents the number of cases and the X-axis stands for the date.

Figure 7 displays the boxplots of the number of cases forecasted between May 31 and June 9, 2020. As shown, the forecasted values are linearly increasing. These values follow the trend of the actual previous values which were stable contrary to the actual values provided between 31 May to 9 June.

**Figure 8:**
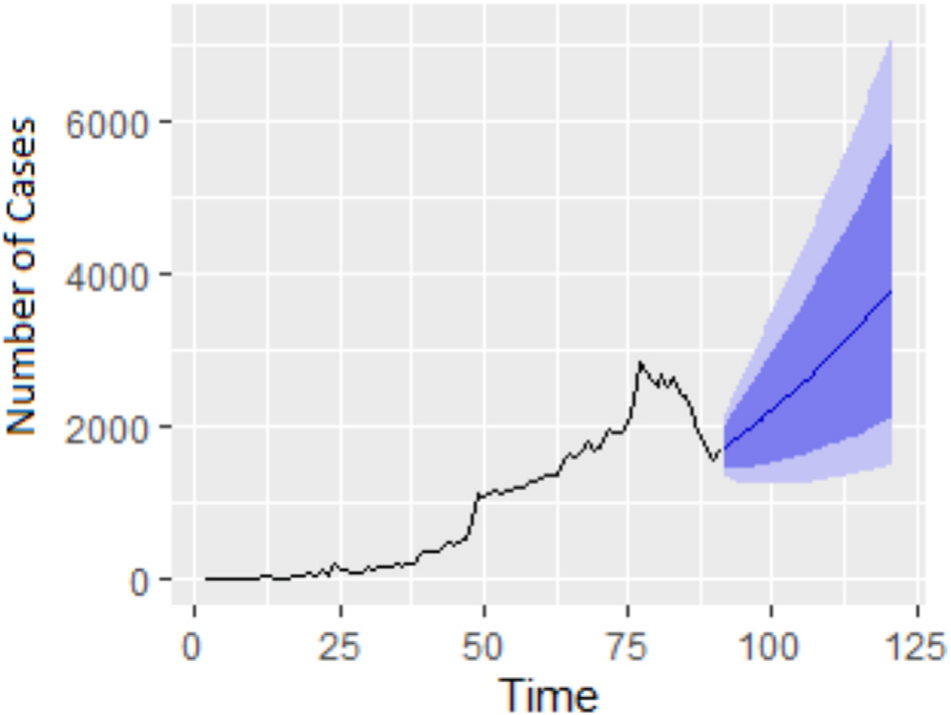
KSA - Number of COVID19 casthe es forecasted using ETS method between May 30 – June 30 (P-Value <0.05). The Y-axis represents the number of cases and the X-axis stands for the time (the number of days).

Table 4 displays the evaluation measures. The RMSE is greater than the MAE with a difference equal to 163.64 which reflects the dissimilarity in the individual errors. This difference is somewhat large because of the unexpected increase of the number of cases in these ten days (31/05 to 09/06). Moreover, the MPE indicates that the average percentage errors between the forecasts and the actual values is about 17%. In other words, the forecasting quality is about 83%. Finally, the ACF1 indicates that the current value is influenced by the previous values (correlation = 70%). Therefore, the five last values couldn't be efficiently predicted since the values between 31/05 and 09/06 achieved 3000 cases whereas the previous values didn't exceed 2000 cases. Figure 8 presents the forecasted values until 30/06/2020. The dark (resp. light) blue color indicates the 80% (resp. 95%) prediction interval. The maximum number of cases is expected to not exceed 4000 cases per day by June 2020 of June 2020. However, the predicted interval (80%) indicated that the forecast values are between 2000 and 5000 cases.

**Table 4:** KSA - Evaluation of ETS model using the testing set.

Measures	RMSE	MAE	MPE	ACF1
Test set	679.3891	515.7540	17.4239	0.7053

### 4.2 Forecasting the number of cases in Brazil

Table 5 shows the details of the Brazil dataset, the minimum and maximum values, the total value, the mean, and the standard deviation. It also includes the size of the training and testing sets. As displayed, there is a large variation between the values (mean and standard deviation are very large).

**Table 5:** Brazil – dataset details.

Total	Min	Max	Mean	Standard Deviation	Training set size	Testing set size
498440	1	33274	5538.222	7246.635	90	10

**Figure 9:**
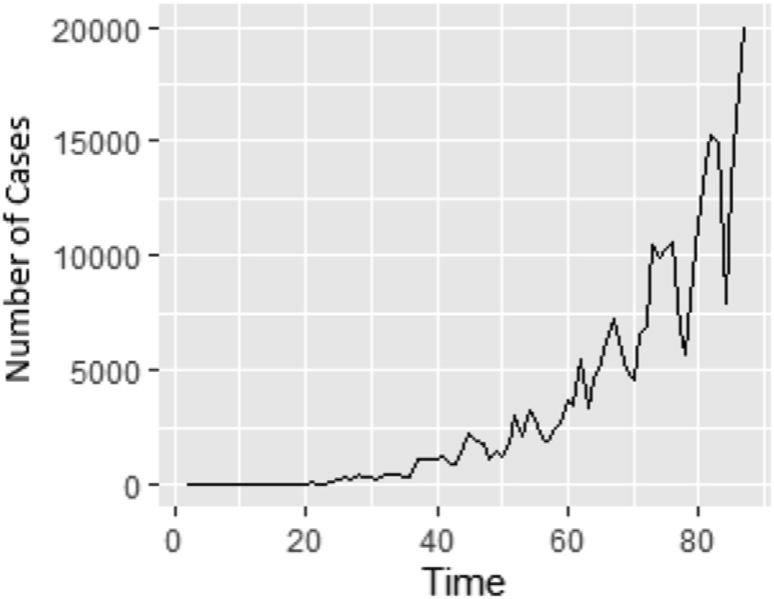
Brazil - The time-series representing number of cases (from Feb 26 to May 21). The Y-axis represents the number of cases and the X-axis stands for the time (the number of days).

The Brazil dataset was collected from February 26 to May 31. The 10-CV was applied to the training set (February 26 to May 21) to find the best parameters’ values for Holt and SES techniques. Then the best selected model was validated using the testing set (May 22–31). Figure 9 displays the time-series of the training set. One can notice that is not stationary. So, the process detailed in the previous section was employed for this dataset. To save space, figures and explanations were omitted.

Table 6 figures out the RMSE from the residuals and the training set, along with the P-Value computed from the Ljung-Box test for the four models. The P-Values for all the models don't indicate a good fit for the data. However, according to [12], the models might be used for forecasting (since the RMSE values from the residuals are insignificant) but their accuracy will be low. So, ETS is selected for forecasting.

**Table 6:** BRAZIL - The p-value and RMSE values for the training set and the residuals using the forecasting methods.

	Drift	SES	Holt	ETS
P-Value	0.00023	0.02659	0.02183	0.02183
Training	4502.002	2155.484	59.2007	39.1186
Residuals	2.2735	2.0479	1.8494	1.8494

**Figure 10:**
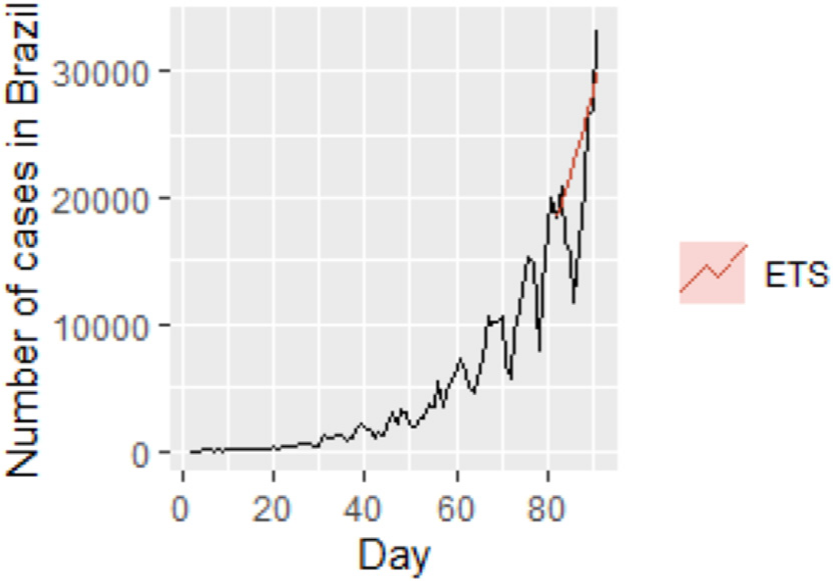
Brazil - COVID19 forecasted number of cases from May 22–31. The Y-axis represents the number of cases and the X-axis stands for the time (the number of days).

Figure 10 presents the number of forecasted cases using the testing set (May 22–31). The perturbation seems to increase with the days. This is because the number of cases from March 2 to May 22 was small and increased slowly, while the number of cases on May 22–31 jumped from 20000 to 30000. In other words, the values of the training model are very different (small) from the values of the testing model (large). So, the forecasted values increased slowly following the training model, while the actual values jumped from 20000 to 30000 cases.

Table 7 indicates that the forecasted values are not very close to the actual values (the black curve). Therefore, the RMSE from the testing set is not low (see Table 7). Moreover, the variance in the individual errors (the difference between RMSE and MAE) achieved 1210.0442 which is large due to the lack of fit of the dataset. The MPE, with a negative result, indicates that the actual values are greater than the forecasted values. Finally, the ACF1 shows the influence of the past values on the future values (about 51%).

**Table 7:** Brazil – Evaluation of ETS model using the testing.

Measures	RMSE	MAE	MPE	ACF1
Test set	5280.021	4069.9768	-21.8686	0.5109847

**Figure 11:**
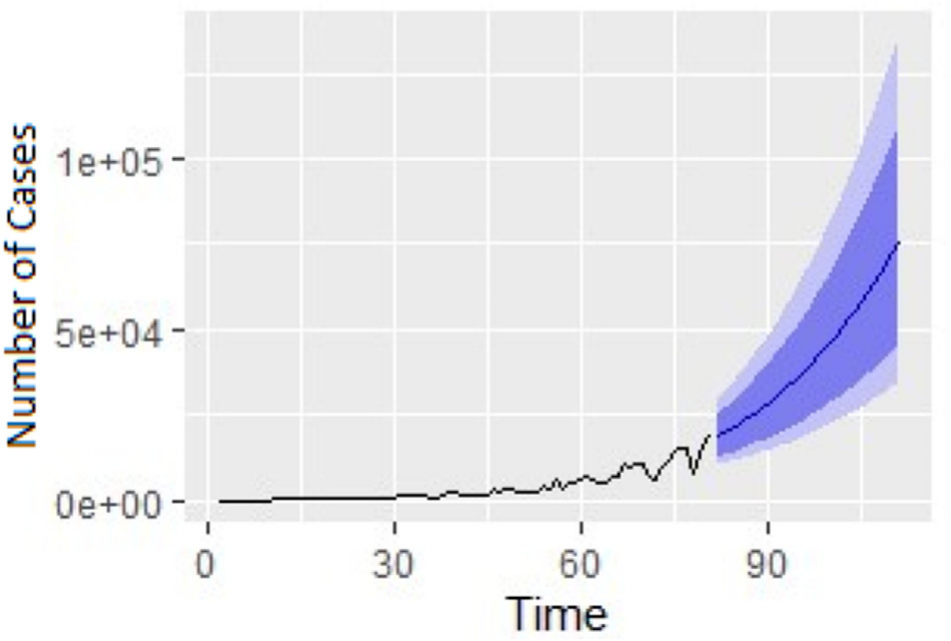
Brazil - COVID19 forecasted number of cases for June 2020***4.3 Forecasting the number of cases in the US.*** The Y-axis represents the number of cases and the X-axis stands for the time (the number of days).

Figure 11 displays the forecasted number of cases for June 2020. The number of cases is still increasing and can be around 10000 and 40000 in June2020. This forecast is inflated because the number of cases in Brazil is high. This result was induced by the fact that the model didn't fit well the data.

Table 8 shows the details of US dataset, the minimum and maximum values, the total value, the mean, and the standard deviation. It also includes the size of the training and the testing sets. Alike Brazil dataset, there is a large variation between the values (mean and standard deviation are very large).

**Table 8:** US – dataset details.

Total	Min	Max	Mean	Standard Deviation	Training set size	Testing set size
1770384	1	48529	13412	13191.53	90	10

**Figure 12:**
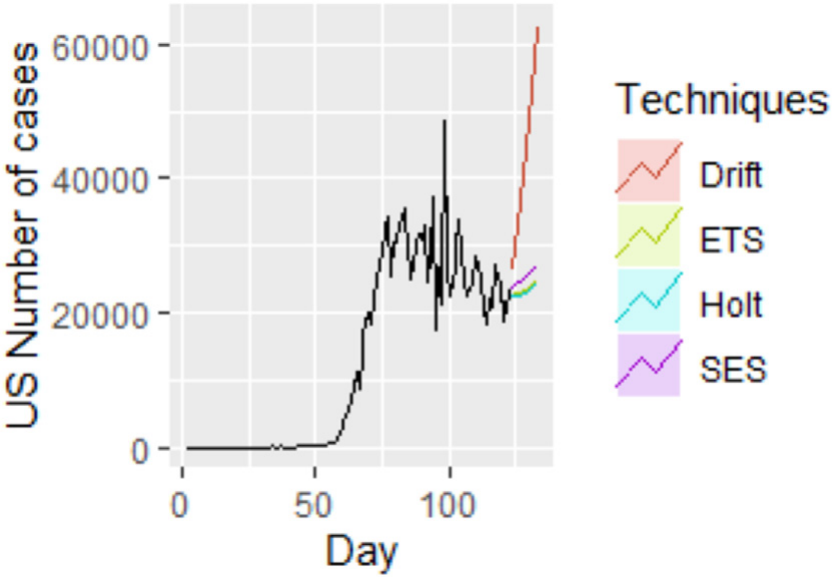
US - Number of COVID19 cases forecasted (testing set May 22–31) using all the methods. The Y-axis represents the number of cases and the X-axis stands for the time (the number of days).

The dataset was collected from January 21 to May 31, divided into the training set (January 21 to May 21) and the testing set (May 22–31). Table 9 exhibits the P-Value for each model, and the RMSE values from both the training set and the residuals. The best model (with the lowest RMSE value) is ETS. Holt model gained the second position. SES yielded the highest RMSE value, and hence, is rejected. These results are confirmed in Figure 12 where the number of cases forecasted for ten days (May 22–31) is displayed.

**Table 9:** US – The P-value and RMSE values for the training set and the residuals using the four forecasting methods.

	Drift	SES	Holt	ETS
P-Value	0.0003	0.245	0.7235	0.5401
Training	28266.38	6274.319	17.2757	4.4497
Residuals	2.846624	2.4880	2.3191	2.2946

Table 10 shows the results of the evaluation measures for ETS and Holt using the testing set (May 22–31). The Holt model is superior to ETS when using the testing set. The ACF1 indicates the correlation between the forecasted and the previous values (0.53 and 0.52 for ETS and Holt respectively). So, the greater ACF1, the smaller the RMSE. Moreover, the variance in the individual errors is about 330.32 and 265.796 for ETS and Holt respectively. Finally, the MPE shows that the actual values are slightly greater than the predicted values.

**Table 10:** US – Evaluation of ETS model using the testing set.

Measures	RMSE	MAE	MPE	ACF1
ETS Testing	3017.577	2687.257	-9.189413	0.5268518
Holt Testing	2735.936	2470.140	-7.235159	0.5177277

Table 11 displays the real values of the number of cases in the US (May 22–31) as well as the forecasted values yielded by Holt and ETS. As seen, the proposed forecasting models are efficient to predict future values based on the provided dataset.

**Table 11:** US - The number of cases forecasted using ETS (May 22–31,2020).

Date	Real Value	Holt	ETS
22-05-2020	25434	22465	22693
23-05-2020	24147	22449	22750
24-05-2020	21236	22481	22848
25-05-2020	20568	22570	22994
26-05-2020	19064	22726	23197
27-05-2020	18910	22957	23460
28-05-2020	18721	23272	23787
29-05-2020	21817	23681	24183
30-05-2020	25337	24191	24650
31-05-2020	23297	24813	25191

**Figure 13:**
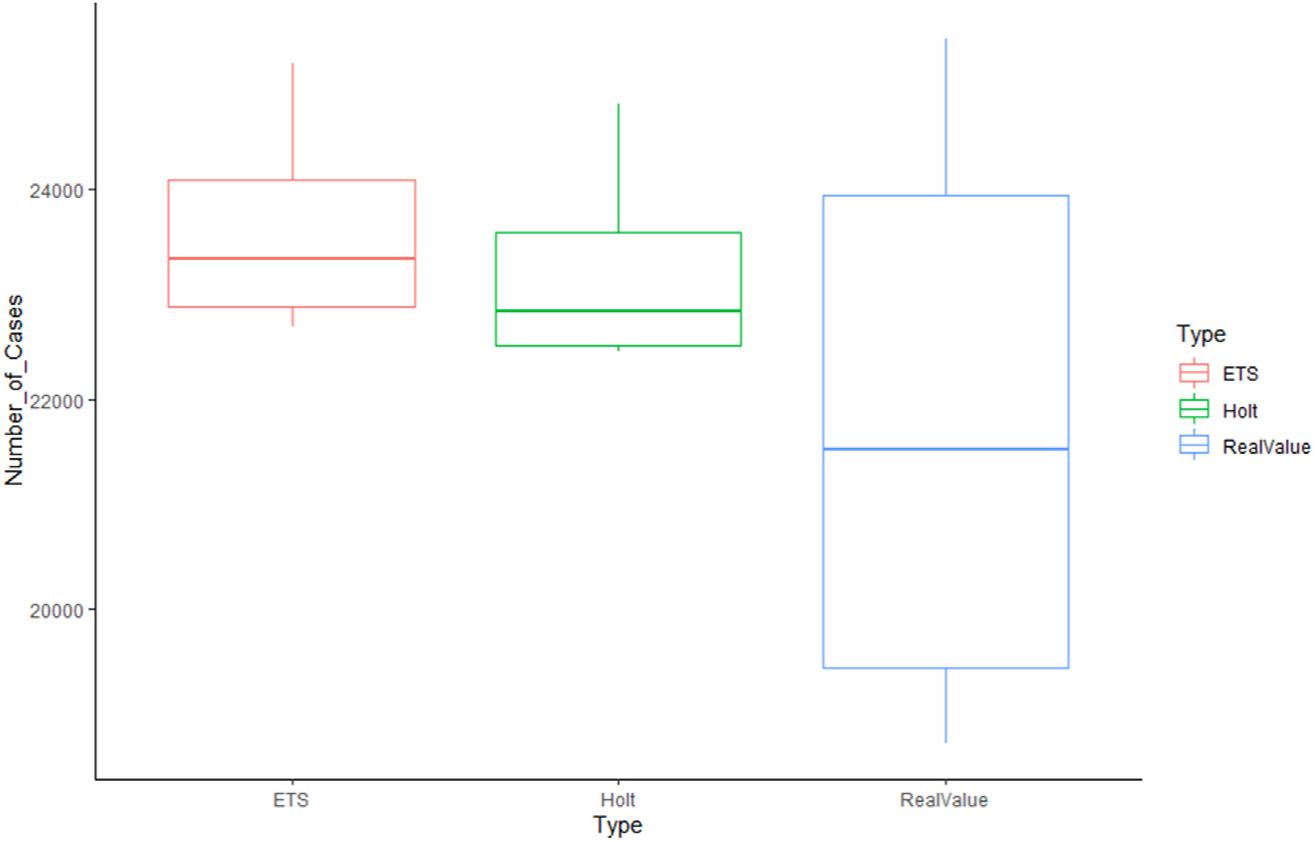
US – Boxplots of the real and the forecasted values of number of cases (May 22–31,2020). The Y-axis represents the number of cases and the X-axis indicates the forecasting methods used and real values.

Figure 13 displays the boxplots of the real and the forecasted number of cases using ETS and Holt between 21 and 30 May. As indicated, the forecasted values range between 22000 and 25000 confirmed cases whereas the real values expand from 18000 to 25000. The real values indicate that the confirmed cases fell (from 25434 in May 22) to 18721 confirmed cases on May 28, to increase to 25337 on May 30. This variability affected the Accuracy of the results.

**Figure 14:**
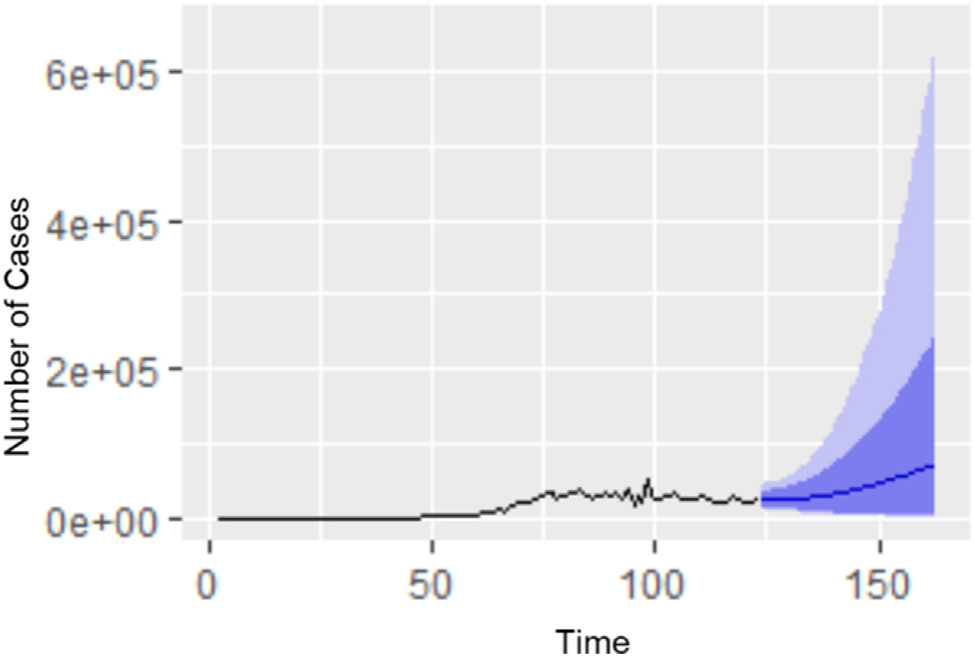
US – the Number of COVID19 cases forecasted (until June 30,2020) using ETS method. The Y-axis represents the number of cases and the X-axis stands for the time (the number of days).

Figure 14 shows the number of cases forecasted in June 2020. Based on the ETS model, the forecasted values are between 10000 and 50000. However, the number of cases might remain high following the prediction intervals. This can be expected since the lock-down is no longer imposed.

### 4.3 Forecasting the number of cases in Spain

Table 12 shows the details of Spain dataset, the minimum and maximum values, the total value, the mean, and the standard deviation. It also includes the size of the training and testing sets. The mean and standard deviation are very large.

**Table 12:** Spain – dataset details.

Total	Min	Max	Mean	Standard Deviation	Training set size	Testing set size
244599	1	9181	2470.697	2529.043	90	10

The Spain dataset was collected from February 01 to May 31 and divided into training (February 01 to May 21) and testing set (May 22–31). As presented in Table13, the Holt and ETS models are selected based on the P-Values (*>* 0*.*05) and the RMSE from both the residuals and the training set. Table 14 sets out the RMSE results from the testing for ETS and Holt models. The ETS outperforms again the Holt model. The negative value of MPE indicates that both models yielded future values lesser than the actual values. Besides, the variance of the individual errors is about 191 and 200 for ETS and Holt respectively, which are not high compared to the Brazil results. However, there is a lack of correlation between the previous the predicted values (about -0.13 and -0.09 for ETS and Holt respectively, see the ACF1).

**Table 13:** Spain - The P-value and RMSE values from the training set and the residuals using the forecasting methods.

	Drift	SES	Holt	ETS
P-Value	0.02715	0.005475	0.2226	0.1047
Training	1979.784	1780.588	293.1407	173.6283
Residuals	897.4764	876.451	0.2785	0.5843

**Table 14:** Spain – Evaluation of ETS and Holt models using the testing set.

Measures	RMSE	MAE	MPE	ACF1
ETS Testing	493.6207	301.7801	-1.581006	-0.1304494
Holt Testing	500.4079	300.5976	-8.598741	-0.09755344

**Figure 15:**
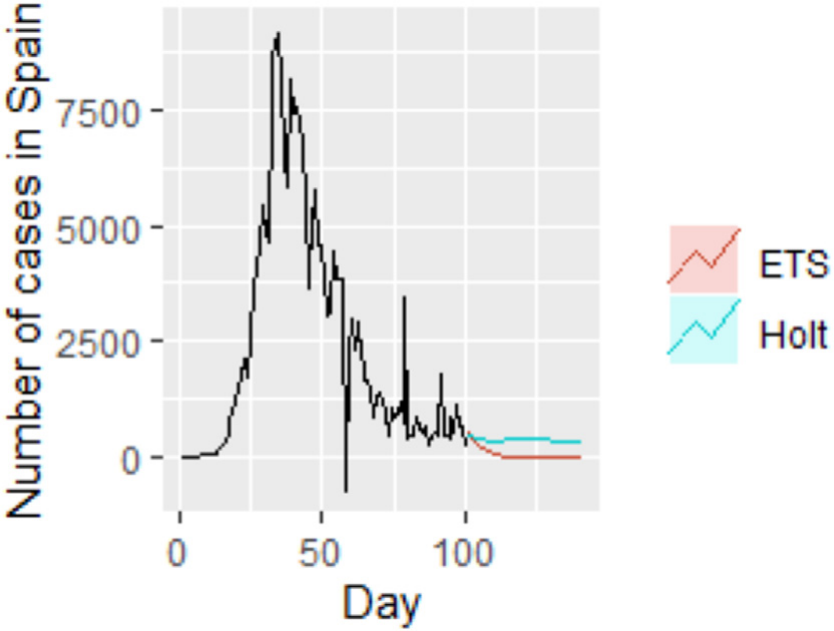
Spain - Number of COVID19 cases forecasted (until June 30,2020) using ETS and Holt methods. The Y-axis represents the number of cases and the X-axis stands for the time (the number of days).

Figure 15 shows the forecasted number of cases for June using both models. The number of cases is expected to decrease. Holt model provides values between 300 and 470, whereas ETS estimates to reach 0 by the end of June.

## 5 Discussion

To sum up, four forecasting case studies have been investigated involving four different countries, KSA, Brazil, US and Spain. All the datasets were preprocessed to make the time-series stationary. With respect to the model validation, the four models were validated to forecast future values (with high P-Value) for KSA case study. While only Holt and ETS (respectively SES, Holt, and ETS) models were validated for the US (respectively Spain) case study. However, even though no model was validated for Brazil case study, the forecasting was performed. The ETS forecasting technique outperformed the three proposed models (considering RMSE) in the four case studies, and Holt achieved better results than Drift and SES. This is because Holt is a type of ETS which represents a Multiplicative error, an Additive Trend, and No seasonality. It is worth to notice that the multiplicative and the additive models (the selected ETS and Holt) result in the same forecast values but with distinct prediction intervals. The validation of the selected model was performed using four evaluation measures (RMSE, MAE, MPE, and ACF1). The forecasting results using the Brazil time-series resulted in a high RMSE (5280) and a high variance of the individual errors (1210) because the model didn't correctly fit the data. The reason behind this result is that the Brazil dataset has high mean and standard deviation (Table 5, mean = 5538 and Standard Deviation = 7247) which reflects high difference between the number cases (in March: 1 confirmed case and in May: 33472 confirmed cases). So, this effect has weakened the forecasting process.

Moreover, the highest ACF1 was found in the KSA case study. This means that there is a strong correlation between the KSA time-series and the number of forecasted cases. The greater the ACF1 the smaller the RMSE. This is not true with US time-series where the ACF1 exceeded 52% but the RMSE achieved 3000 (for ETS). This effect is interesting when large random values can be expected in the time-series (See Table 8, mean = 13412, Standard Deviation = 13992). Alike Brazil dataset, the US dataset reflects a high variability (with a minimum number of cases = 1 in March and the maximum number of cases = 48529 in May). Furthermore, the forecasted number of cases for Spain, US, and Brazil were less than the actual values (MPE*<* 0) due to some unexpected large values appeared in the testing set. The ETS model succeeds in forecasting future values for the four countries even if the results of Brazil are somewhat inflated. The 80% and 95% prediction intervals provided a gap between low and high values due to an unexpected change and lack of consistency in the datasets. This resulted in a prediction uncertainty, but the obtained forecasts are promising. The obtained results showed that by the end of June, the number of cases (per day) in KSA, Brazil, and US continues to increase to reach approximately 4000–5000 cases per day in KSA, 10000–40000 cases in Brazil, 10000 and 50000 in the USA. Whereas it is expected to fall to 300–470 cases in Spain by June 2020.

### 5.1 Comparison study

#### 5.1.1 Comparison study with state-of-the-art studies

The KSA dataset described above (March 02 to May 31) was used in [1]. Table 15 displays the RMSE results obtained by ETS and Holt (the proposed models), and ARIMA [1]. As seen, the ETS and Holt outperformed ARIMA. This result is due to not only the performance of ETS and Holt but also to 1)-the parameter setting applied in this study to find the best values of Holt's parameters and 2)-the preprocessing performed on the time-series. This comparison study demonstrated the efficiency of the proposed study. The proposed models significantly enhanced the forecasting results by about 88%.

**Table 15:** Comparison study results for KSA.

Models	Proposed Holt	Proposed ETS	ARIMA
RMSE	20.8855	18.4361	107.5396
P-Value	0.6521	0.5812	<0.05

Moreover, two studies forecasted the total number of cases in US from February 2020 to July 2^nd^, 2020 in [16], and from February 2020 to July 10^th^, 2020 in 0+. Both datasets were collected and processed as explained above. Table 16 displayed the results of [15] as well as the proposed Holt and ETS models. As, displayed, MAPE obtained by ETS and Holt are clearly smaller than the results obtained by Stacked LSTM, Bi-directional LSTM, and Convolution LSTM. Both proposed models outperformed the three DL models in forecasting the cumulative number of cases. However, the Convolution LSTM is a competitive model as its result is not far from the results yielded by Holt and ETS. In the contrast, the results of Bi-directional and Stacked LSTM were enhanced by 4.72% and 8.06% using ETS and Holt respectively.

**Table 16:** US – Results of the Comparison study with [15].

Models	Proposed Holt	Proposed ETS	Stacked LSTM	Bi-directional LSTM	ConvLSTM
MAPE	1.94	1.94	10.00	6.66	2.00

Table 17 shows the forecasted values of the total number of cases between 26 June and 02 July 2020 using the proposed Holts and ETS models, and the SutteARIMA [16]. The three models yielded values close to the actual values. However, SutteARIMA is better than ETS and Holt in terms of MAPE. The authors in [16] did not use another metric to further compare the results.

**Table 17:** US – Results of Comparison study with [16].

Date	Actual	Proposed Holt	Proposed ETS	SutteARIMA
26/06/2020	2552956	2463825	2463824	2544732
27/06/2020	2596537	2504119	2504116	2590888
28/06/2020	2637077	2545431	2545425	2632477
29/06/2020	2681811	2587936	2587928	2671055
30/06/2020	2727853	2631818	2631807	2711798
01/07/2020	2779953	2677265	2677251	2755128
02/07/2020	2837189	2724475	2724456	2803729
MAPE		3.601921	3.602240	0.00539

#### 5.1.2 Comparison study with other algorithms

This section presents the comparison with some ML algorithms. Firstly, H2O's AutoML was used. It consists of the automation of the different phases of ML (data preprocessing, training, testing, validation, parameter setting, etc) [29]. It includes several ML algorithms such that Gradient Boosting Machine (GBM), Generalized Linear Model (GLM), Distributed Random Forest (DRF), eXtremely Randomized Trees (XRT), and Stacked Ensemble (using the one of only the best models of each kind of these algorithms).

H2O's AutoML requires more than one independent variable to predict the dependent variable. For this, KSA dataset was used with three independent variables (Date, Number of Deaths, and Number of Recovered) to predict the number of Cases. The same training and testing sets (used in the proposed models, from March 2^nd^ to May 30^th^ for the training, and for May 31^st^ to June 9^th^ for the testing) were used in this comparison. Table 18 presents the RMSE values from the training set using the H2O's AutoML algorithms. Among AutoML proposed algorithms, the best model yielding the lowest RMSE value is XRT. However, the ETS outperformed the best model XRT.

**Table 18:** KSA - RMSE values from the training set using H2O's AutoML and ETS.

Models – H2O's AutoML	RMSE
XRT	288.0577
GBM	298.1039
DRF	300.0999
Stacked Ensemble	210.3833
Proposed Model: ETS	18.4361

Table 19 figures out the results of ETS and XRT using the testing set. Again, ETS outperforms XRT in terms of RMSE and MAE.

**Table 19:** KSA - Evaluation of ETS and XRT models using the testing set.

Model	RMSE	MAE
XRT	908.3867	721.6342
ETS	679.3891	515.7540

Secondly, XGBoost was used. XGBoost (or eXtreme Gradient Boosting) is an enhanced version of distributed Gradient Boosted Decision Tree (GBDT) library. It affords parallel tree boosting. It is a well-known library for classification and regression [30]. In this comparison, the four datasets (KSA, Brazil, Spain, and the US) were utilized (considering only the “Date” variable and previous values of Cases). The training and the testing sets are similar to what was used above.

Table 20 displays the values of the RMSE, MAE, and MPE for the XGBoost and the proposed models using the testing set for each dataset. As displayed, ETS (respectively Holt) performed better than XGBoost for all the datasets (respectively, US and Spain).

**Table 20:** Comparison study - Evaluation of XGBoost and the proposed models using the testing set for each dataset.

Dataset	Model	RMSE	MAE	MPE
KSA	ETS	679.3891	515.7540	17.4239
	XGBoost	822.3128	610.1452	17.72728
Brazil	ETS	5280.021	4069.9768	-21.8686
	XGBoost	6190.753	4918.1	-4.469476
US	ETS	3017.577	2687.257	-9.189413
	Holt	2735.936	2470.140	-7.235159
	XGBoost	3272.734	2734.444	-11.26654
Spain	ETS	493.6207	301.7801	-1.581006
	Holt	500.4079	300.5976	-8.598741
	XGBoost	497.9066	317.1167	8.140828

AutoML and XGBoost are mainly dedicated to large datasets with a considerable number of features (independent variables) which is not the case for the datasets used in this study. The four datasets are small and the predicted results (the number of cases) mainly depend on their-previous values. Hence, the comparison study with the ML algorithms showed that the proposed statistical methods are more appropriate to forecast the number of Covid 19 confirmed cases.

### 5.2 Forecasting the number of deaths in KSA

In this section, three research questions were answered to investigate the number of deaths in KSA. The dataset was collected from March 02 to May 30.

1) Can the number of deaths be predicted based on both the numbers of cases and recovered?

The dataset was normalized before being transformed to time-series using Eq. (1).

(1) $$z_{i}=\frac{x_{i}-\min\left(x\right)}{\max\left(x\right)-\min\left(x\right)}$$

Where is the data point at time t.

The prediction of the number of deaths was performed using the Multiple Linear Regression (MLR). The obtained MLR equation is presented in Eq. (2).

(2) Death = 0.07492 + 0.37909 × Cases +0.24325 × Recovered

**Figure 16:**
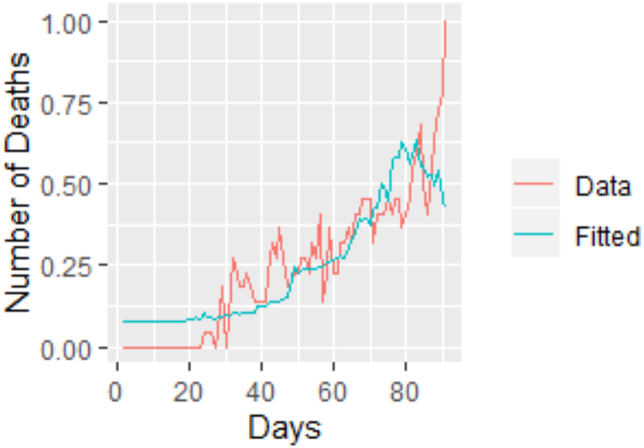
KSA - Representation of the Multiple Regression Model. The Y-axis represents the number of deaths and the X-axis stands for the time (the number of days).

**Figure 17:**
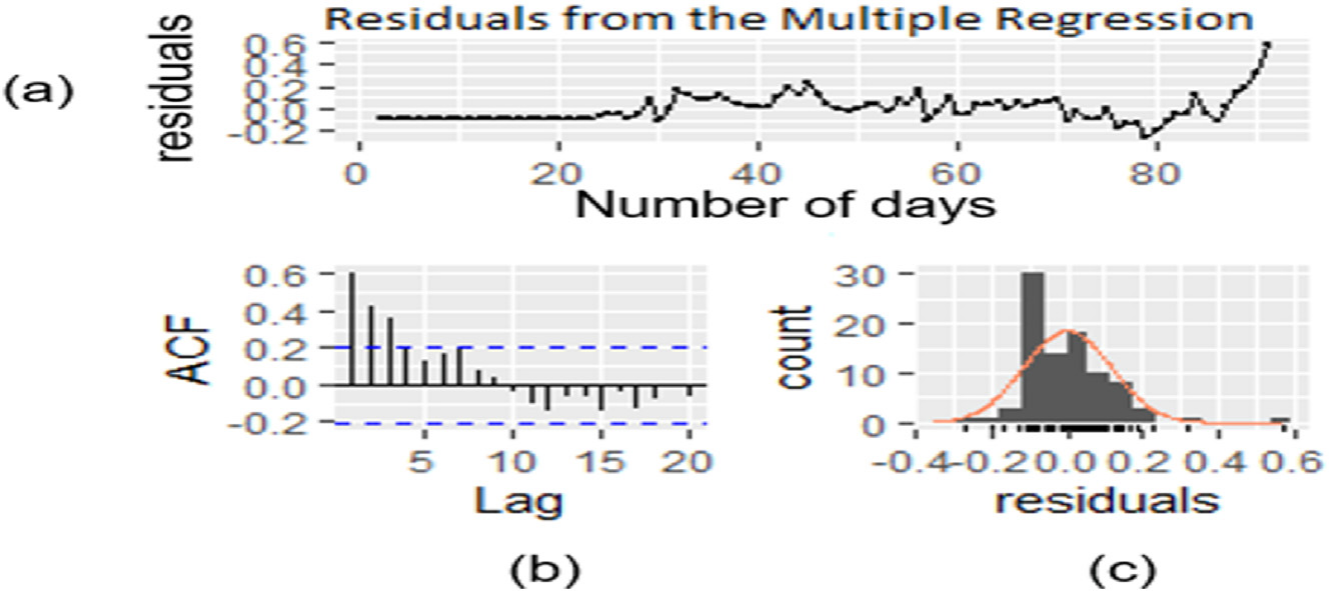
KSA – The residuals from the MLR model. The Y-axis represents the residuals values (in panel (a)), the ACF values (in panel (b)), and number count (in panel (c)). While the X-axis indicates the number of days (in (a)), the lags (in (b)), and the residuals (in (c)).

The predictor “Cases” is the most influencing variable. There is one death for every two cases and two recovered. Table 21 presents the standard error, the t- and P-Values for the intercept, Cases and Recovered variables. The Cases and Recovered variables have a strong relationship with the Death variable due to a small P-Value (*<* 0*.*05). The P-Value is useful when studying the effect of each predictor but is not particularly useful for forecasting [7]. Figure 16 shows the actual values compared to the fitted values after applying MLR. To show how well the MLR model fits the data, the coefficient of determination (R squared) was calculated. R-squared achieved 0.7044. So, the model does a good job as it explains 70.44 % of the variation in the dataset. Also, the residual standard error of this model is equal to 0.1173 which is insignificant. After the regression model being fitted, the residuals are plotted to check whether the time-series can be used to forecast future values. The results are displayed in Figure 17. The residuals are not uncorrelated (3 lags exceed the blue line in the ACF plot) and don't follow the Normal distribution, with a no-null mean (see the histogram). The P-Value (=8.273e-08 computed from the Breusch-Godfrey) indicates that the model doesn't fit the entire data. Consequently, forecasting the Death number cannot be applied using the numbers of Cases and recovered because the conditions required for the MLR model are not met.

2) Can the number of deaths be predicted based on the number of cases?

**Table 21:** KSA - Parameters and evaluation measures obtained from MLR for the “cases” and “recovered” variables.

	Std. Error	t value	P-Value
Intercept	0.01843	4.066	0.000105
Cases	0.07181	5.279	9.44e-07
Recovered	0.08043	3.025	0.003273

The same process discussed above was applied using Linear Regression (LR). The regression equation is presented in Eq. (3).

(3) Death = 0.06055 + 0.56109 × Cases

**Figure 18:**
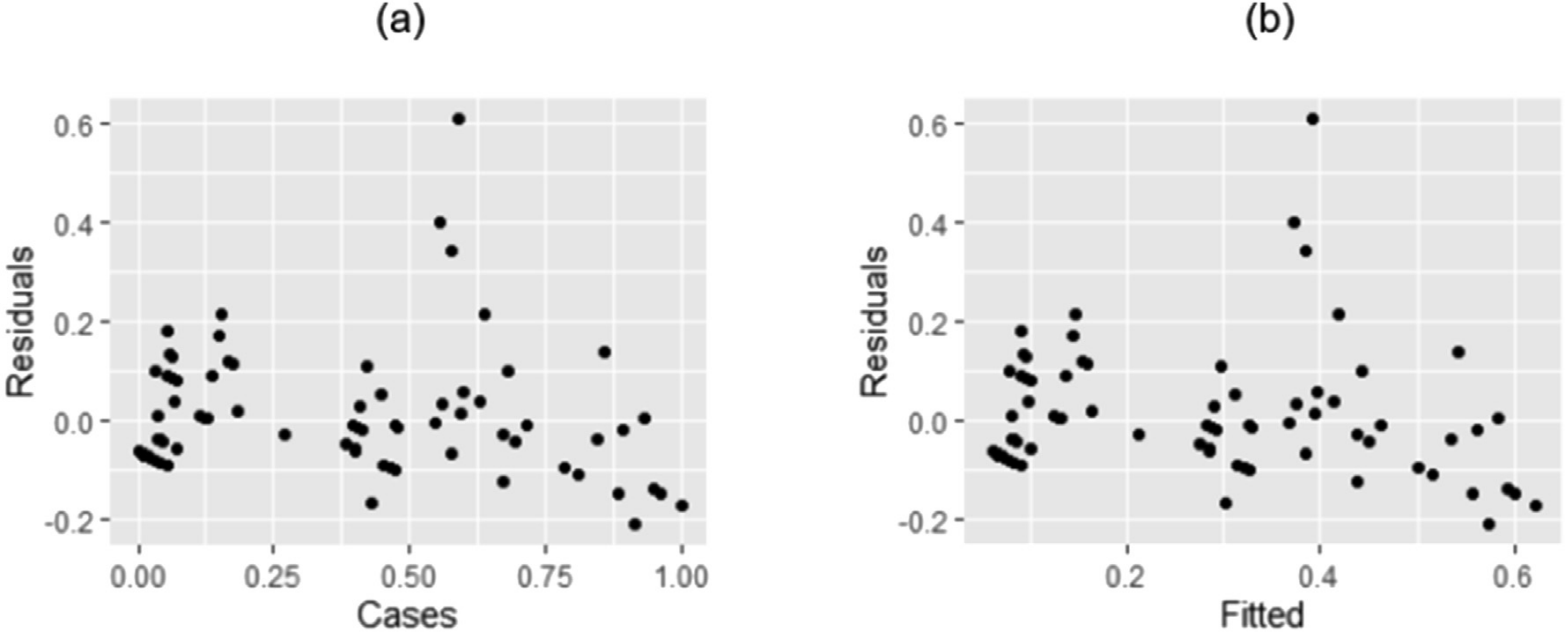
Scatter plot of the residuals (represented in the Y-axis in both panels (a) and (b)) against “Cases” variable (indicated in the X-axis in panel (a)) and the fitted model (indicated in the X-Axis in panel (b)).

The predictor variable “cases” is the most influencing variable. Its P-Value is < 2e − 16 and R-squared reached 0.677. The residuals are uncorrelated and possess information that might be useful when forecasting future values (small P- Value equals to 2.226e-08). They have a linear pattern with the predictor variable (left) and the fitted model (right) as displayed in Figure 18. Thus, forecasting the number of Deaths cannot be applied using the number of Cases.

3) Can the number of deaths be forecasted (alone)?

In the following, the four forecasting techniques were used to forecast the number of deaths. The dataset was divided into training (March 2 until May 30) and testing (May 31 to June 9). The same process was followed. Prior to forecast future values, the residuals of each model were investigated. The Ljung-Box test yielded a high P-Value (see Table 22) which confirms the effectiveness of the fitted model obtained by the four techniques. Furthermore, the RMSE results from the residuals, for all the models, are small and less than the RMSE values from the training set. SES outperformed the three models, and the Drift model performed better than the Holt and the ETS models (Table 22). The four models yielded promising results even though the SES is the best one. So, all the models were kept for the validation and the forecast phases.

**Table 22:** KSA Death - The P-value and RMSE values from the training set and the residuals using the forecasting methods.

	Drift	SES	Holt	ETS
P-Value	0.1254	0.208	0.1122	0.3021
Training	3.229823	2.690445	3.938953	4.055494
Residuals	1.27837	1.226366	1.169782	1.212663

**Figure 19:**
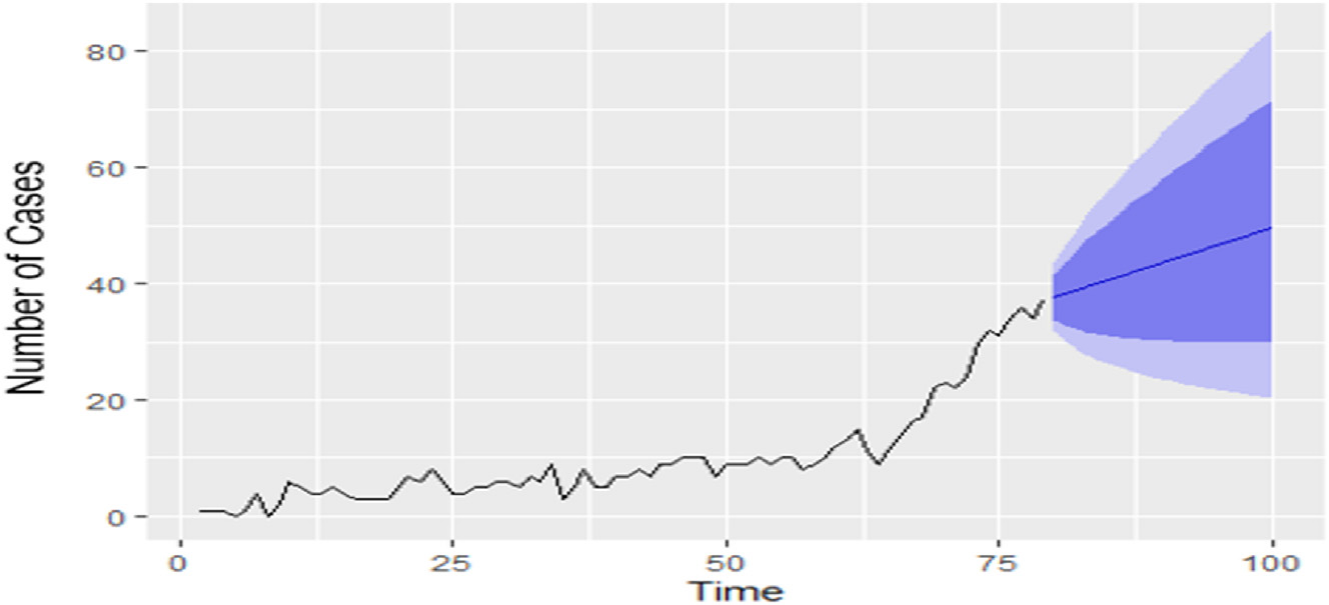
KSA Death – Number of COVID19 deaths forecasted (until June 30) using Drift model. The Y-axis represents the number of cases and the X-axis stands for the time (the number of days).

Table 23 presents the results of four evaluation measures using the testing set. Drift is the best model (RMSE = 7.3758). The variance of the individual errors reached 1.11 which is a small value. The forecasted values are greater than the actual values (MPE *>* 0). The forecasted and the previous values are correlated (ACF1 = 66%). Figure 19 displays the number of deaths forecasted (until June 30) using the Drift model. The number of deaths per day is expected to range between 30 and 84 (see the prediction interval) with an average of 50 per day by the end of June 2020. So, the pandemic is still subsisting in KSA.

**Table 23:** KSA Death – Evaluation of the four models using the testing set.

	RMSE	MAE	MPE	ACF1
Drift	7.375841	6.267739	18.17841	0.6591618
SES	10.785257	9.484588	29.04443	0.68395988
Holt	10.974308	10.102519	31.69407	0.6644047
ETS	11.905337	10.729140	33.27339	0.6843702

## 6 Conclusion

In conclusion, the recentCOVID-19 pandemic has had its tolls in a lot of countries around the world. Thus, scientists in different fields are working on designing various models to build a better understanding of the situation and propose valid solutions. Prediction and forecasting models are vital to provide a well-validated view of the pandemic situation in the future and consequently help in designing the proper solutions. Our work is an additional block added to this field. It aimed to predict the number of COVID-19 cases (resp. death) for four countries including KSA, USA, Spain, and Brazil (resp. KSA). Forecasting was performed using time-series and four techniques (Drift, SES, ETS, Holt).

The experiments went through five stages. First, time-series stationary was validated using well-known techniques and tests. Then, the residuals of each model were investigated to ensure that the models can be applied to forecast new values. Following that, the best forecasting model was selected based on RMSE. Lastly, the best model was validated using four evaluation measures (RMSE, MAE, MPE, ACF1). The forecast was successfully performed with the prediction intervals 85%–90%. The obtained results estimated that in June 2020 the per-day number of cases in KSA reaches around 5000 cases and reaches around 10000–40000 in Brazil. While it is predicted to reach 200–370 confirmed cases in Spain, and 10000–50000 in the US. However, the forecasted number might not exactly reflect the actual numbers of cases/deaths because they can be affected by the different imposed events like the lockdown and the curfew, and their release. The comparison study showed that the proposed ETS and Holt models outperformed ARIMA, the three variants of LSTM Deep Learning techniques. However, both models are competitive with SutteARIMA. Moreover, ETS and Holt also outperformed the ML algorithms provided by AutoML and XGBoost. Furthermore, the last case study showed that the number of deaths can also be forecasted using the same models. The results indicate that the number of deaths in KSA can reach an average of 50 per day by the end of June 2020.

To sum up, the present study showed the effectiveness of the Exponential Smoothing techniques in forecasting the spread of COVID19 disease. The well-known and old statistical models (ETS and Holt) can surpass ARIMA, Drift, SES, LSTM deep learning technique, XGBoost, and AutoML algorithms. This can be achieved with a good preprocessing of the time-series and best parameter setting (for Holt). ETS and Holt are competitive forecasting models that deserve to be more investigated in any forecasting problem. The existing studies involved many statistical, ML, and DL techniques but not ETS and Holt. The forecast of the COVID19 parameters used only the past confirmed cases/deaths numbers without requiring additional factors. This model can be applied to forecast an ongoing changing situation of any kind of disease, and not just COVID-19 pandemic, by providing sufficient data.

The main limitation of this work is that the prediction intervals provided a gap between low and high values due to an unexpected change and lack of consistency in the datasets. The forecasted values highly depend on the previous actual values. So, if the last actual values raised suddenly, the forecasted values would follow the trend of the actual values. The sudden change will affect the results and increase the gap between the forecasted and existing values. Hence, the increased values of the metrics. To minimize the evaluation metrics results, it is recommended to forecast few days or a limited period (for example between 3 and 6 days) instead of forecasting a whole month.

This study could be extended to explore Deep Learning technique (other than LSTM). Moreover, the different inflected events like the lockdown, the curfew, and their release make change to the datasets. So, the inclusion of these factors in the dataset could be of interest. Furthermore, forecasting the Spread Growth Rate and the Case Fatality Rate (discussed in [31]) using the proposed models could help the authority in decision making.

## Declarations

### Author contribution statement

Souad Larabi-Marie-Sainte, Sara Shaheen: Conceived and designed the experiments; Performed the experiments; Analyzed and interpreted the data; Wrote the paper.

Sawsan Alhalawani, Khaled Mohamad Almustafa, Tanzila Saba, Fatima Nayer Khan, Amjad Rehman: Conceived and designed the experiments; Analyzed and interpreted the data; Wrote the paper.

### Funding statement

This work was supported by Prince Sultan University.

### Data availability statement

Data associated with this study is available at https://www.ecdc.europa.eu/en/publications-data.

### Declaration of interests statement

The authors declare no conflict of interest.

### Additional information

No additional information is available for this paper.
